# Supplementary material for: Integrin alpha11 is an Osteolectin receptor and is required for the maintenance of adult skeletal bone mass
Source: eLife. 2019 Jan 11;8:e42274. doi: 10.7554/eLife.42274 (PMC6349404; doi:10.7554/eLife.42274)
Supplement: Supplementary file 1. — Stem and progenitor cell populations were isolated from mouse bone marrow by flow cytometry using the listed markers. Lineage (Lin) markers used to isolate Lineage negative cell populations were CD2, CD3, CD5, CD8, Ter119, Gr-1, and B220. [file elife-42274-supp1.docx]

**Supplementary file 1. Markers used for the flow cytometric isolation of bone marrow cell populations.** Stem and progenitor cell populations were isolated from mouse bone marrow by flow cytometry using the listed markers. Lineage (Lin) markers used to isolate Lineage negative cell populations were CD2, CD3, CD5, CD8, Ter119, Gr-1, and B220.

| **Full description** | **Abbreviation** | **Marker Used to Isolate** | **References** |
| --- | --- | --- | --- |
| Bone Marrow Cells | BMC | Ter119^-^ |  |
| Endothelial Cells | Endo | VE-cadherin^+^, CD45^-^, Ter119^-^ |  |
| LepR^+^ Cells | LepR^+^ | LepR^+^, CD45^-^, Ter119^-^, CD31^-^ | (Zhou et al., 2014) |
| Osteoblasts | OB | *Col2.3*-*GFP*^+^ | (Kalajzic et al., 2002) |
| Common Myeloid Progenitors | CMP | Lin^-^, c-kit^+^, Sca1^-^, CD34^+^, CD16/32^-^ | (Akashi et al., 2000) |
| Granulocyte and Monocyte Precursors | GMP | Lin^-^, c-kit^+^, Sca1^-^, CD34^+^, CD16/32^+^ | (Akashi et al., 2000) |
| Megakaryocyte and Erythroid precursors | MEP | Lin^-^, c-kit^+^, Sca1^-^, CD34^-^, CD16/32^-^ | (Akashi et al., 2000) |
| Common Lymphoid Progenitors | CLP | Lin^-^, c-kit^low^, Sca1^low^, CD127^+^, CD135^+^ | (Kondo et al., 1997) |
| CD3+ T Cells | CD3+ | Ter119^-^, CD3^+^ |  |
| Erythroid cells | Erythroid | Ter119^+^, CD71^+^ |  |
| Myeloid cells | Myeloid | Mac-1^+^, Gr-1^+^ |  |
| c-kit+ Progenitors | LSK | Lin^-^, c-kit^+^, Sca1^+^ | (Okada et al., 1992) |
| Multipotent Progenitors | MPP | Lin^-^, c-kit^+^, Sca1^+^, CD150^-^, CD48^-^ | (Kiel et al., 2008) |
| Hematopoietic Stem Cells | HSC | Lin^-^, c-kit^+^, Sca1^+^, CD150^+^, CD48^-^ | (Kiel et al., 2005) |
| B220+ B Cells | B220+ | Ter119^-^, B220^+^ |  |
| Pro-B Cells | Pro-B | B220^+^, CD43^+^, CD24^+^, IgM^-^ | (Li et al., 1996) |
| Pre-pro-B Cells | Prepro-B | B220^+^, CD43^+^, CD24^-^, IgM^-^ | (Li et al., 1996) |
